# The influence of aspect markers and tense on the action-sentence compatibility effect in Mandarin action sentence comprehension

**DOI:** 10.1371/journal.pone.0340298

**Published:** 2026-01-23

**Authors:** Ning Fan, Hongkai Zhu, Zhiwei Cai

**Affiliations:** 1 College of education, Hebei University, Baoding, Hebei, China; 2 College of teacher education, Baoding University, Baoding, Hebei, China; University of Missouri Columbia, UNITED STATES OF AMERICA

## Abstract

The Action-sentence Compatibility Effect (ACE) suggests that action sentences comprehension is based on embodied mental simulation. In studies of English, aspect markers and tense has been shown to influence ACE; however, their effects remain unclear in Mandarin. This study utilized Mandarin action sentences with single objects and employed the classic sentence sensibility judgment paradigm to investigate the ACE for concrete and abstract action sentences under different aspect marker (Experiment 1 and 2) or future tense (Experiment 3) conditions across three experiments. The results showed that ACE occurred for both concrete and abstract action sentences in the progressive aspect, perfective aspect, and future tense. Action simulation during sentence comprehension was unaffected by the type of aspect markers and future tense or the concreteness/abstractness of the sentences. These findings suggest that the comprehension of Mandarin action sentences relies on the mental simulation of verbs. Moreover, aspect markers and future tense in Mandarin do not influence the mental simulation process, and Mandarin action sentences comprehension may be centered around the mental simulation of the verb.

## Introduction

From the perspective of embodied cognition, language comprehension is a process of mental simulation guided by grammar [1–3]. The Action-sentence Compatibility Effect (ACE), discovered by Glenberg and Kaschak [4], serves as critical experimental evidence supporting this view. ACE manifests as faster reaction times when the action direction implied in a sentence aligns with subsequent button-press directions, and slower reaction times when they conflict. For example, participants who read a sentence describing an action moving away from the body, such as “You delivered the pizza to Andy,” responded faster with a forward arm movement. Conversely, reading a sentence describing an action moving toward the body, such as “Andy delivered the pizza to you,” resulted in quicker responses with a backward arm movement. Similar effects were observed even when concrete objects like “pizza” were replaced with abstract concepts such as “story,” as in “Liz told you the story” [4].

Research on ACE provides evidence for the role of embodied mental simulations in language comprehension processes [5–7]. This perspective aligns with the Perceptual Symbol Systems theory [8], which proposes that our understanding of concepts is not purely abstract or symbolic, but based on sensory and motor experiences. According to this theory, when people read a verb like “push” or “pull,” they automatically activate perceptual details associated with the action—such as the direction of movement, physical effort, or typical body posture. In other words, sentence comprehension is thought to involve recreating or “simulating” these experiences in the mind, drawing on memory and prior interaction with the physical world.

However, studies on ACE across different languages, despite using similar materials or paradigms, have yielded inconsistent results due to variations in grammatical structures and lexical semantic features [9,10]. One influential account of ACE is the Indexing Hypothesis [4], which proposes that comprehending action-related sentences involves three sequential stages: indexing (identifying relevant objects or entities in the sentence), affordance extraction (activating associated sensorimotor features), and meshing (integrating these features under the constraints of grammar to simulate the described action).

Studies have shown that grammatical elements such as syntactic role (e.g., agents and patients), can modulate the mental simulation of action sentences [7,11]. Among such grammatical cues, aspect markers and tense play crucial role in encoding both the temporal structure and the dynamic status of the actions. This leads to an important question: do these markers—by signaling whether an action is ongoing, completed, or impending—influence how readers mentally simulate actions during sentence comprehension?

Research on English has demonstrated that aspect markers can affect action simulation. For instance, Bergen and Wheeler found that changing a sentence in the progressive aspect (e.g., John is closing the drawer) to the perfective aspect (e.g., John has closed the drawer) eliminated the previously observed ACE [12]. Similarly, Liu and Bergen found that ACE occurred only when participants comprehended sentences in the progressive aspect, but not in the perfective aspect [13]. Bergen and colleagues argued that sentences in the perfective aspect emphasize the completed state of events, thereby halting mental simulation of core actions described in the event [12]. These findings suggest that aspect markers may influence ACE: the use of the perfective marker to indicate completion emphasizes the resulting state of the action [14,15], potentially rendering action simulation unnecessary for sentence comprehension.

Similar findings have also been reported in studies on tense. For example, Madden and Zwaan, using a sentence-picture matching paradigm, showed that after reading sentences in the past tense, participants responded faster to images representing completed actions than to those depicting ongoing actions [16], indicating they did not mentally represent the internal event structure but instead represented the completed state of the action.

The above findings suggest that syntax plays a critical role in sentence comprehension. On one hand, syntax “combines” or “integrates” the simulated content of nouns and verbs; on the other, it adjusts higher-order features of mental simulation. For example, syntax dictates what perspective to adopt, in what order to simulate, and whether an action is simulated [1,4,12].

The temporal location of events is encoded differently across languages, with English relying primarily on tense and Mandarin predominantly on aspect [17]. In English, tense and aspect are exprressed through verb inflections (e.g., -s, -ed) and auxiliary constructions (e.g., be + V-ing, have + V-ed), with strict word order.By contrast, Mandarin lacks overt verb inflections and instead marks aspect through adverbs or particles placed before or after the verb (e.g., *正在/zhèngzài/* for progressive aspect; *已经...了/yǐjīng...le/* for perfective aspect). Temporal regerence in Mandarin is typically conveyed through temporal adverbs or inferred from context. Nevertheless, some linguistic studies suggest that Mandarin may exhibit a future vs. non-future tense distinction [18,19], and that tense information can be encoded syntactically despite the absence of explicit tense morphology. These structural characteristics suggest that Mandarin may encode temporal information differently from English. Importantly, Chinese aspect markers operate at a semantic–syntactic interface, where both syntactic and semantic cues are functional in online processing. This dual role underscores that Mandarin aspectual marking is not simply a substitute for tense but reflects distinct mechanisms of temporal interpretation.

Given these differences, it is possible that Mandarin aspect markers interact with syntactic cues and contextual information to influence how actions are mentally simulated during sentence comprehension [20,21]. This raises important questions: do aspect markers in Mandarin shape the integration of described actions in mental simulations? And similar to English, would sentences describing non-current actions, such as those with future reference, reduce or eliminate the ACE? Research on Mandarin ACE therefore offers critical insights into how language-specific temporal marking systems modulate embodied language processing, thereby enriching cross-linguistic perspectives on the relationship between grammar, meaning, and simulation.

Additionally, Fischer and Zwaan suggested that many studies using double-object structures inherently imply directional information [22]. For concrete action sentences (hereafter referred to as “concrete sentences”), ACE may arise from both the semantic and syntactic attributes of the actions, whereas for abstract action sentences (hereafter referred to as “abstract sentences”), ACE may primarily be influenced by syntax during the action integration stage. Research on whether individuals perform mental simulation during the comprehension of abstract sentences has yielded inconsistent results [13,23]. To avoid confusion between semantics and syntax in ACE and further investigate the effect of aspect markers on mental simulation in concrete and abstract action sentences, this study used Mandarin action sentences with single objects.

Following previous research [12], the study employed the sentence sensibility judgment paradigm and selected two aspect markers—the progressive aspect and perfective aspect—to examine how aspect markers influence mental simulation during the comprehension of Mandarin action sentences. Additionally, the study explored whether sentences with future tense involve the same mental simulation process. Experiment 1 investigated the effect of progressive aspect and perfective aspect on ACE in concrete sentences, while Experiment 2 examined the same effect in abstract sentences. Experiment 3 further explored whether ACE occurs in sentences with future tense sentences for both concrete and abstract action sentences. If aspect markers and future tense influence mental simulation during sentence comprehension, ACE will occur only in progressive sentences, indicating that Mandarin aspect markers also shift the focus of mental simulation during sentence comprehension. Conversely, if ACE is not affected by aspect marker and future tense, it would suggest that Mandarin action sentences comprehension relies more on verb-centered mental simulation. Furthermore, if abstract sentences also exhibit ACE, it would imply that the mental simulation process during sentence comprehension is based on the perceptual symbol representations of lexical items, rather than the characteristics of syntactic structure.

## Experiment 1: The influence of aspect markers on mental simulation during the comprehension of concrete action sentences in Mandarin

### Predictions

Based on previous findings [12], Experiment 1 aimed to examine whether aspect markers influence ACE during the comprehension of concrete action sentences in Mandarin. Specifically, we predicted that the progressive aspect would elicit a significant ACE, as it describes ongoing actions that are more likely to evoke embodied simulations. In contrast, the perfective aspect, which denotes completed actions, was expected to weaken or eliminate ACE due to reduced relevance of ongoing motor representation.

### Design

Forty right-handed native Chinese-speaking participants were recruited from a university. All participants had normal or corrected-to-normal vision. The study was approved by the Ethics Committee of Affiliated Hospital of Hebei University (protocol code: HDFY-LL-2021–178). All participants provided written informed consent prior to the experiment in accordance with the Declaration of Helsinki. The same ethical procedure was applied to the subsequent experiments. The recruitment period for participants in the three experiments was from November 2021 to November 2022.

Experiment 1 is a mixed experimental design with 2 (aspect markers: progressive aspect/perfective aspect) × 2 (semantic direction: describing action toward the body/ describing action away from the body) × 2 (key position: near the body/far from the body). The key position is a between-subjects variable, while aspect markers and the semantic direction of the verb are within-subjects variables. Each level of the between-subjects variable (key position) included 20 participants, with all participants exposed to all within-subjects conditions. The dependent variables are accuracy and response time.

### Meterials

The experimental materials consisted of simple declarative sentences with third-person name nouns as subjects. First, 200 verb-object phrases, such as “打开抽屉/ dǎkāi chōutì/ open a drawer”, were compiled. Thirty university students not participated in the formal experiment rated the concreteness and directionality of the phrases using a 7-point Likert scale (1 = very concrete/toward the body, 4 = uncertain, 7 = very abstract/away from the body) and rated familiarity using a 5-point Likert scale (1 = very unfamiliar, 3 = uncertain, 5 = very familiar). Based on these ratings, 40 sets of materials were selected, including 20 concrete phrases describing actions toward the body and 20 describing actions away from the body. Person name nouns, temporal adverbs, or auxiliary words were added to form the final experimental sentences, which were then rated for sensibility using a 5-point Likert scale (1 = very unreasonable, 3 = uncertain, 5 = very reasonable). The progressive aspect marker is “正在/ zhèngzài/ is...”, and the perfect aspect marker is “已经...了/ *yǐjīng...le*/ has...”. For example, “小明正在打开抽屉/ Xiǎo Míng zhèngzài dǎkāi chōutì/ Xiao Ming is opening a drawer.” was a concrete action sentence with the progressive aspect marker, describing action toward the body, while “王敏已经退还了定金/ Wáng Mǐn yǐjīng tuìhuán le dìngjīn/ Wang Min has returned the deposit.” was a concrete action sentence with the perfective aspect, describing action away from the body. A total of 40 unreasonable sentences (e.g., 小慧正在烹制会议/ Xiǎo Huì zhèngzài pēngzhì huìyì/ Xiaohui is cooking a meeting) were compiled as fillers to balance participants’ responses, with equal representation of the progressive aspect and present perfective aspect.

The four groups of verb-object phrases were matched for verb concreteness, stroke count, verb familiarity, and noun familiarity, as well as sentence sensibility. MANOVA results showed no significant differences across groups (*ps* > 0.05). Details of the material matching results are presented in Table 1.

**Table 1:** Matching Results for Experimental Materials in Experiment 1 (*M* ± *SD*).

	Concreteness	Directionality	Stroke count	Verb familiarity	Noun familiarity	Present continuous familiarity	Present perfect familiarity
Concrete-Toward	1.47 ± 0.20	1.84 ± 0.38	32.30 ± 6.73	4.79 ± 0.11	4.82 ± 0.09	4.37 ± 0.15	4.35 ± 0.27
Concrete-Away	1.51 ± 0.20	5.51 ± 0.31	31.80 ± 6.65	4.79 ± 0.07	4.78 ± 0.21	4.47 ± 0.17	4.49 ± 0.08

### Procedures

Our experimental procedure followed previous related studies [12]. Before the experiment begins, participants are informed that they must quickly and accurately determine whether the sentence is reasonable by pressing keys according to the experiment’s instructions. Yellow labels are placed on the “**A**” key, red labels on the “**H**” key, and blue labels on the “ **’** “ key of a standard computer keyboard. The three keys are aligned in a straight line, with a 4-key gap between adjacent keys. During the experiment, the computer keyboard is rotated 90° counterclockwise, making it perpendicular to its normal orientation. The yellow key, which is closer to the participant, is positioned on the side of the keyboard that is nearer, while the blue key, which is farther away, is positioned on the opposite side. The keyboard is adjusted to ensure it is within the participant’s field of view, and sufficient practice is provided so that the participant can perform the keypress operation without needing to turn their head or look down.

Participants were randomly assigned to two response conditions (Yes-Far/Yes-Near). Each participant read 40 randomly presented reasonable sentences (10 progressive-toward, 10 progressive-away, 10 perfective-toward, 10 perfective-away) and 40 unreasonable sentences. In the “Yes-Far” condition, participants were required to press the blue key, which was farther from the body, if they judged the sentence to be reasonable, and press the yellow key, which was closer to the body, if they judged the sentence to be unreasonable. In the “Yes-Near” condition, the key responses were reversed. After each key press to judge a sentence, participants had to place their finger back on the red key. During the experiment, participants used their right index finger for key responses. Each trial began with a fixation point (“+”), and the fixation point disappeared when the red key was pressed. The sentence then appeared at the center of the screen, and participants were required to make a judgment on the sentence’s plausibility within 5 seconds. After pressing the key, the sentence disappeared, followed by a 1-second blank screen between trials. The entire experiment lasted 20–30 minutes, with three breaks of 3 minutes each.

### Analysis

The experimental data were preprocessed in Excel and then statistically analyzed using *RStudio*. Participants with accuracy below 80% were first excluded, followed by trials with errors or those that deviated more than 3 standard deviations from the average value of each condition. Sentence accuracy was calculated using the formula: (number of correctly judged sentences/ total number of sentences) × 100%. The total number of excluded data points was less than 5% of all data.

We used the lme4 package to build linear mixed-effects models (LMMs) for analyzing sentence response times [24]. Following the recommendations of Barr et al. [25], we first constructed a full model with the maximal random-effects structure. The fixed effects included the main effects of aspect markers, semantic direction, and key position, as well as their interactions. The random effects included random intercepts for participants and items, and by-participant random slopes for aspect markers and semantic direction.

If the full model failed to converge, we simplified the random-effects structure step by step according to the procedure suggested by Barr et al.: first removing correlations among random slopes, and then removing random slopes for participants or items. We compared candidate models using the Akaike Information Criterion (AIC) and Bayesian Information Criterion (BIC), and when a model with more random effects did not perform significantly better than a more parsimonious one, we adopted the simpler model as the final model.

After determining the optimal model, we used the *lmerTest* package to assess the significance of fixed effects [26], the *effectsize* package [27] to compute standardized *β* values and confidence intervals for fixed effects, and the emmeans package to calculate marginal means and conduct post-hoc comparisons [28], with multiple comparisons corrected using the Tukey method.

### Results

Two participants with accuracy below 80% were excluded from the experiment. The average response times and accuracy under different conditions are shown in Table 2.

**Table 2:** Average reaction time and accuracy in Experiment 1 (*M* ± *SD*).

		Reaction time		Accuracy	
Aspect markers	Key position	Toward	Away	Toward	Away
Progressive aspect	Yes-Near	1452 ± 278	1593 ± 360	94.21 ± 8.38	93.68 ± 10.11
	Yes-Far	1574 ± 391	1588 ± 339	96.84 ± 5.82	98.42 ± 5.82
Perfective aspect	Yes-Near	1577 ± 357	1674 ± 426	96.32 ± 5.97	96.84 ± 7.49
	Yes-Far	1645 ± 420	1584 ± 351	95.26 ± 6.97	97.89 ± 4.19

The model testing results showed that the full model, which included all fixed effects, random intercepts for participants and items, as well as by-participant random slopes for sentence type and semantic direction, failed to converge (AIC = 22322, BIC = 222412). Under convergence conditions, and according to the ΔAIC/ΔBIC criteria [29], Model 3— which included all fixed effects and random intercepts for participants and items (AIC = 22312, BIC = 222370)—was superior to Model 2, which included random intercepts for participants and items and by-participant random slopes for sentence type (AIC = 22314, BIC = 222382). Therefore, Model 3 was identified as the optimal model.

The random-effects results of the optimal model showed that the variance of participant intercepts was 117086 (*SD* = 342), the variance of item intercepts was 10973 (*SD* = 104), and the residual variance was 221121 (*SD* = 470). These results indicate that both individual differences and item differences contributed to the overall variability in reaction times, although the residuals remained the largest source of variance.

The fixed-effects results of the optimal model showed that the main effect of aspect markers was not significant (*β* = 0.140, *t* = 1.213, *p* = 0.230, *CI* = [−0.087, 0.367]); the main effect of semantic direction was not significant (*β* = 0.084, *t* = 0.734, *p* = 0.466, *CI* = [−0.141, 0.310]); and the main effect of key position was not significant (*β* = −0.204, *t* = −0.989, *p* = 0.328, *CI* = [−0.610, 0.201]).

The interaction between aspect markers and semantic direction was not significant (*β* = −0.163, *t* = −1.003, *p* = 0.320, *CI* = [−0.483, 0.156]); the interaction between aspect markers and key position was not significant (*β* = 0.112, *t* = 0.947, *p* = 0.344, *CI* = [−0.120, 0.345]); and the interaction between semantic direction and key position was significant (*β* = 0.235, *t* = 1.985, *p* = 0.047, *CI* = [0.003, 0.468]). Post-hoc comparisons showed that in the “Yes-Near” condition, the mean reaction time for sentences describing action toward the body (emmean = 1527, *SE* = 85) was significantly shorter (*t* = −2.807, *p* = 0.007) than that for sentences describing action away from the body (emmean = 1663, *SE* = 85). In contrast, in the “Yes-Far” condition, the difference between sentences describing action toward the body (emmean = 1614, *SE* = 85) and away from the body (emmean = 1616, *SE* = 85) was not significant (*t* = −0.034, *p* = 0.973). Finally, the three-way interaction among aspect markers, semantic direction, and key position was not significant (*β* = −0.017, *t* = −0.099, *p* = 0.921, *CI* = [−0.345, 0.312]).

### Discussion

The experimental results showed that in sentences describing concrete actions, both present continuous and present perfect aspect markers exhibited ACE. That is, when participants pressed the key with their bent arm directed toward their body, they responded faster to sentences describing action toward the body and slower to sentences describing action away from the body. These results differ from previous related studies. Bergen and Wheeler, using the same experimental paradigm, found no ACE for present perfect sentences [12]. They suggested that perfect aspect marker sentences emphasize the completed state of an event, which shuts down the mental simulation of the core action described in the sentence. The results of this experiment suggest that the cognitive mechanisms for understanding sentences in Mandarin and English may differ. In the process of understanding Mandarin action sentences, the aspect markers during the integration phase does not affect participants’ mental simulation of the actions described in specific action sentences.

## Experiment 2: The influence of aspect markers on mental simulation during the comprehension of abstract action sentences in Mandarin

### Predictions

Experiment 2 investigated whether aspect markers modulate ACE in abstract action sentences, which lack concrete motor representations. If ACE is still observed in abstract sentences with the progressive aspect but not with the perfective aspect, it would suggest that Mandarin aspect markers influence mental simulation regardless of the concreteness of the sentence content.

### Design

A total of 40 right-handed native Chinese-speaking participants with normal or corrected-to-normal vision were recruited from a university.

The experimental design same as Experiment 1. Each level of the between-subjects variable (key position) included 17 participants, with all participants exposed to all within-subjects conditions.

### Meterials

Based on the evaluation results of the materials from Experiment 1, 40 abstract action sentences (10 progressive-toward, 10 progressive-away, 10 perfective-toward, 10 perfective-away) were selected. For example, “小赵正在博得同情/Xiǎo Zhào zhèngzài bó dé tóngqíng/Xiao Zhao is gaining sympathy” is a progressive aspect sentence describing action moving toward the body, whereas “小勇已经泄露了机密/Xiǎo Yǒng yǐjīng xièlùle jīmì/Xiao Yong has already leaked the secret” is a perfective aspect sentence describing an action moving away from the body. MANOVA results showed no significant differences across groups (*ps* > 0.05). The matching results of the materials are shown in Table 3.

**Table 3:** Matching Results for Experimental Materials in Experiment 2 (*M ± SD*).

	Concreteness	Directionality	Stroke count	Verb familiarity	Noun familiarity	Present continuous familiarity	Present perfect familiarity
Abstract toward	4.72 ± 0.44	1.94 ± 0.31	33.80 ± 4.93	4.76 ± 0.19	4.80 ± 0.09	4.35 ± 0.19	4.51 ± 0.23
Abstract away	4.93 ± 0.43	5.39 ± 0.44	34.95 ± 6.48	4.80 ± 0.07	4.79 ± 0.11	4.48 ± 0.31	4.44 ± 0.19

Similarly, 40 sentences with the same aspect marker as the formal materials but semantically unreasonable were included to balance the participants’ key responses. The sentences were equally divided between progressive aspect and perfective aspect.

### Procedures

The experimental procedure same as Experiment 1.

### Analysis

The experimental analysis methods same as Experiment 1.

### Results

Four participants with accuracy below 80% were excluded from Experiment 2. Descriptive results are shown in Table 4.

**Table 4:** Average reaction time and accuracy in experiment 2 (*M ± SD*).

		Reaction time		Accuracy	
Aspect markers	Key position	Toward	Away	Toward	Away
Progressive aspect	Yes-Near	1616 ± 624	1583 ± 510	87.37 ± 33.25	94.15 ± 23.53
	Yes-Far	1798 ± 765	1651 ± 664	89.88 ± 30.25	94.67 ± 22.52
Perfective aspect	Yes- Near	1609 ± 547	1835 ± 691	92.11 ± 27.04	85.19 ± 35.62
	Yes-Far	1758 ± 745	1772 ± 656	94.67 ± 22.52	90.59 ± 29.29

The model testing results showed that the full model, which included all fixed effects, random intercepts for participants and items, as well as by-participant random slopes for sentence type and semantic direction, failed to converge (AIC = 19862, BIC = 19945). Under convergence conditions, and according to the ΔAIC/ΔBIC criteria [29], Model 3— which included all fixed effects and random intercepts for participants and items (AIC = 19845, BIC = 19911)—was superior to Model 2, which included random intercepts for participants and items and by-participant random slopes for sentence type (AIC = 19857, BIC = 19925). Therefore, Model 3 was identified as the optimal model.

The random-effects results of the optimal model showed that the variance of participant intercepts was 159100 (*SD* = 399), the variance of item intercepts was 13283 (*SD* = 115), and the residual variance was 20927 (*SD* = 458). These results indicate that both individual differences and item differences contributed to the overall variability in reaction times, although the residuals remained the largest source of variance.

The fixed-effects results of the optimal model showed that the main effect of aspect markers was not significant (*β* = −0.021, *t* = −0.175, *p* = 0.862, *CI* = [−0.254, 0.213]); the main effect of semantic direction was not significant (*β* = −0.200, *t* = −1.682, *p* = 0.0974, *CI* = [−0.433, 0.033]); and the main effect of key position was not significant (*β* = −0.348, *t* = −1.501, *p* = 0.141, *CI* = [−0.803, 0.107]).

The interaction between aspect markers and semantic direction was not significant (*β* = 0.232, *t* = 1.379, *p* = 0.173, *CI* = [−0.098, 0.561]); the interaction between aspect markers and key position was not significant (*β* = 0.069, *t* = 0.591, *p* = 0.555, *CI* = [−0.160, 0.298]); and the interaction between semantic direction and key position was significant (*β* = 0.286, *t* = 2.456, *p* = 0.014, *CI* = [0.058, 0.515]). Post-hoc comparisons showed that in the “Yes-Near” condition, the mean reaction time for sentences describing action toward the body (emmean = 1563, *SE* = 98) was significantly shorter (*t* = −2.959, *p* = 0.004) than that for sentences describing action away from the body (emmean = 1713, *SE* = 98). In contrast, in the “Yes-Far” condition, the difference between sentences describing action toward the body (emmean = 1757, *SE* = 41) and away from the body (emmean = 1705, *SE* = 41) was not significant (*t* = 1.002, *p* = 0.320). Finally, the three-way interaction among aspect markers, semantic direction, and key position was not significant (*β* = 0.083, *t* = 0.504, *p* = 0.615, *CI* = [−0.241, 0.407]).

### Discussion

The results of Experiment 2 found that in Mandarin abstract action sentences, both progressive aspect and perfective aspect exhibited the ACE, and this effect primarily occurred in responses involving the approach motion. These findings are consistent with some previous studies [4,23,30], but inconsistent with other studies [12,31]. On one hand, this suggests that the understanding of Mandarin abstract action sentences also requires mental simulation of actions. On the other hand, it indicates that the tense markings in Mandarin, which differ from those in English, have different effects on the mental simulation of actions in abstract action sentences. Experiment 3 will further investigate whether the ACE still occurs in future sentences.

## Experiment 3: The influence of future tense on mental simulation during the comprehension concrete and abstract action sentences in Mandarin

### Predictions

Experiment 3 investigated whether the ACE occurs in future-tense sentences in Mandarin, and whether this effect differs between concrete and abstract action descriptions. Since future-tense sentences describe actions that have not yet occurred, they may be less likely to evoke embodied mental simulations. If the ACE is absent in future-tense conditions, this would suggest that temporal distance weakens the mental simulation process during sentence comprehension. Conversely, if ACE is still observed, it would indicate that lexical concreteness alone can trigger embodied simulation, regardless of tense. Furthermore, if the ACE emerges only under concrete action sentence conditions, this would suggest that the influence of future tense on ACE is modulated by the concreteness of action descriptions.

### Design

Forty right-handed native Chinese-speaking participants with normal or corrected-to-normal vision were recruited from a university.

The experiment followed a 2 (sentence type: concrete/abstract) × 2 (semantic direction: toward the body/away from the body) × 2 (key direction: Yes-Far/Yes-Near) mixed design, with key direction as a between-subjects variable. Each level of the between-subjects variable (key position) included 20 participants, with all participants exposed to all within-subjects conditions.

### Meterials

The sentence materials were primarily derived from Experiment 1 and 2, with the aspect markers changed to future (将要/jiāng yào/will). A total of 40 reasonable sentences were included, consisting of 20 concrete action sentences (10 toward, 10 away) and 20 abstract action sentences (10 toward, 10 away), with an equal number of sentences describing action toward or away from the body. Additionally, 40 unreasonable future sentences were included as fillers. The matching of materials in each group was tested using MANOVA (*ps* > 0.05), as shown in Table 5.

**Table 5:** Matching Results for Experimental Materials in Experiment 3 (*M* ± *SD*).

	Concreteness	Directionality	Stroke count	Verb familiarity	Noun familiarity	Sentence sensibility
Concrete-Toward	1.37 ± 0.20	1.80 ± 0.41	32.17 ± 6.93	4.85 ± 0.05	4.87 ± 0.05	4.34 ± 0.21
Concrete-Away	1.54 ± 0.24	5.52 ± 0.20	30.33 ± 5.42	4.80 ± 0.06	4.84 ± 0.13	4.33 ± 0.11
Abstract-Toward	4.93 ± 0.38	2.05 ± 0.34	34.92 ± 4.83	4.82 ± 0.07	4.81 ± 0.10	4.12 ± 0.19
Abstract-Away	5.12 ± 0.23	5.26 ± 0.47	33.42 ± 6.99	4.80 ± 0.06	4.75 ± 0.10	4.12 ± 0.14

### Procedures

The experimental procedure same as Experiment 1.

### Analysis

The experimental analysis methods same as Experiment 1.

### Results

The accuracy of all participants in this experiment was above 80%. Descriptive results are shown in Table 6.

**Table 6:** Average reaction time and accuracy in Experiment 3 (*M* ± *SD*).

		RT (*ms*)		ACC (*%*)	
Sentence type	Key direction	Toward	Away	Toward	Away
Concrete sentences	Yes-Near	1285 ± 254	1399 ± 315	98.50 ± 3.66	98.00 ± 4.10
	Yes-Far	1420 ± 325	1400 ± 327	98.00 ± 4.10	99.00 ± 3.08
Abstract sentences	Yes-Near	1355 ± 307	1397 ± 300	97.50 ± 5.50	97.00 ± 5.71
	Yes-Far	1489 ± 436	1361 ± 364	97.50 ± 4.44	96.50 ± 8.13

The model testing results showed that the full model, which included all fixed effects, random intercepts for participants and items, as well as by-participant random slopes for sentence type and semantic direction, failed to converge (AIC = 23382, BIC = 23546). Under convergence conditions, and according to the ΔAIC/ΔBIC criteria [29], Model 3— which included all fixed effects and random intercepts for participants and items (AIC = 23452, BIC = 23511)—was superior to Model 2, which included random intercepts for participants and items and by-participant random slopes for sentence type (AIC = 23454, BIC = 23524). Therefore, Model 3 was identified as the optimal model.

The random-effects results of the optimal model showed that the variance of participant intercepts was 94499 (*SD* = 307), the variance of item intercepts was 7740 (*SD* = 88), and the residual variance was 169835 (*SD* = 412). These results indicate that both individual differences and item differences contributed to the overall variability in reaction times, although the residuals remained the largest source of variance.

The fixed-effects results of the optimal model showed that the main effect of sentence type was not significant (*β* = 0.142, *t* = 1.275, *p* = 0.207, *CI* = [−0.076, 0.360]); the main effect of semantic direction was not significant (*β* = −0.072, *t* = −0.651, *p* = 0.517, *CI* = [−0.029, 0.145]); and the main effect of key position was not significant (*β* = −0.267, *t* = −1.302, *p* = 0.199, *CI* = [−0.669, 0.135]).

The interaction between sentence type and semantic direction was not significant (*β* = −0.154, *t* = −0.978, *p* = 0.332, *CI* = [−0.462, 0.154]); the interaction between sentence type and key position was not significant (*β* = −0.032, *t* = −0.279, *p* = 0.780, *CI* = [−0.256, 0.192]); and the interaction between semantic direction and key position was significant (*β* = 0.300, *t* = 2.632, *p* = 0.009, *CI* = [0.076, 0.523]). Post-hoc comparisons showed that in the “Yes-Near” condition, the mean reaction time for sentences describing action toward the body (emmean = 1333, *SE* = 75) was significantly shorter (*t* = −2.435, *p* = 0.018) than that for sentences describing action away from the body (emmean = 1431, *SE* = 75). In contrast, in the “Yes-Far” condition, the difference between sentences describing action toward the body (emmean = 1479, *SE* = 75) and away from the body (emmean = 1402, *SE* = 75) was not significant (*t* = 1.897, *p* = 0.062). Finally, the three-way interaction among sentence type, semantic direction, and key position was not significant (*β* = −0.081, *t* = 0.501, *p* = 0.617, *CI* = [−0.236, 0.398]).

### Discussion

The results of Experiment 3 found that in Mandarin future-tense sentences, both concrete and abstract action sentences exhibited the ACE, and the future tense did not affect participants’ mental simulation of the actions. This further suggests that the different aspect markers in Mandarin and English sentence comprehension processes have different effects on mental simulation.

## General discussion

This study investigates the ACE for concrete and abstract action sentences under different aspect marker (Experiment 1 and 2) or future tense (Experiment 3). The experimental results indicated that readers’ mental simulation of actions during the understanding of Mandarin action sentences was not affected by aspect markers or the concrete/abstract sentence. That is, in both concrete and abstract action sentences, whether in the present progressive aspect, perfective aspect, or future tense, the ACE was present. This suggests that participants mentally simulate the action of the verb when understanding both concrete and abstract action sentences, and the embodied cognitive process of action sentence comprehension is psychologically realistic. The different aspect markers in Mandarin and English lead to differences in the manifestation of the ACE, highlighting those syntactic differences between languages should be considered in studies of embodied cognitive mechanisms during sentence comprehension.

According to the indexing hypothesis, sentence action simulation goes through three stages: the indexing and extraction affordance phase, which is the embodied mode of language realization, and the integration phase, where syntax retains its role in the integration of actions [4]. Studies in English have shown that aspect markers and tense affects the ACE, with researchers suggesting that those markers modulate the mental simulation of action in the language comprehension process by adjusting the state of the action [1,2,4,12,32]. For example, the progressive aspect emphasizes that the action is ongoing, while the perfective aspect emphasizes that the action is completed. The experiment by Bergen and Wheeler found that progressive aspect sentences produced a significant ACE [12], while perfective aspect sentences did not exhibit this effect. Similarly, Madden and Zwaan found in their sentence-picture matching paradigm that after presenting a sentence in the past tense, participants responded faster to images depicting the completed state of an event than to those depicting the ongoing state [16], suggesting that participants represented the event’s completion rather than its ongoing nature. These results demonstrate that aspect markers and tense in English have a clear impact on the embodied mental simulation during sentence comprehension.

However, Mandarin is a language that emphasizes meaning rather than grammatical structure, and there are differences between Mandarin and English in terms of tense [17,33] and syntax [34]. A large body of research has explained the differences between languages in terms of sentence processing mechanisms from both behavioral and neural perspectives [20,21,35,36], which may be the reason for the differing results between this study and previous English studies. In English, aspect markers and tense are primarily realized through inflectional changes in verb form (e.g., V-ed, V-s, be+doing, have+done), which serve as clear syntactic cues for temporal processing during sentence comprehension. In contrast, Mandarin expresses aspect and tense mainly through auxiliary markers or aspectual particles (e.g., “了/le”, “过/guò”, “着/zhe”, “正在/zhèngzài”) attached to verbs. Mandarin syntax is comparatively more flexible, relying not solely on linear word order but also on semantic and contextual cues, which may influence how temporal information is mentally represented and processed.

These differences may alter the process of mental simulation, leading to the absence of an effect of aspect markers and tense information in this study. In English, verb inflection not only provides temporal information but also aspectual information, which may make readers more sensitive to the completion state of the action when interpreting verbs, thereby weakening the intensity of mental simulation. As a result, the ACE disappears when the reader makes judgments after understanding the sentence. Some English studies have found that the ACE in sentence comprehension only appears after the verb in the sentence, not at the end of the sentence [36]. In Mandarin action sentences, verbs do not undergo inflectional changes. Instead, aspect and tense information is conveyed through additional temporal expressions and aspect markers. Since the verb itself remains morphologically unchanged across different temporal contexts, the action simulation triggered by the verb may remain stable at the lexical level. The ACE, therefore, may only weaken once the aspectual information has been fully processed and integrated into the overall sentence meaning. This suggests that in Mandarin, mental simulation of actions may not occur solely at the verb level but may emerge during or after sentence-level integration. The influence of action simulation may diminish only after the aspectual context of the verb is completely understood.

The current evidence does not allow us to determine whether aspectual markers are consistently integrated into readers’ mental simulations of described actions. This speculation is mainly based on related findings from research in English. For examle, Van Dam and Desai found in their research using English sentences that the ACE appeared in the left-right direction when participants understood sentences with third-person subjects (e.g., “He threw the microphone”) due to the syntactic spatial representation [7]. However, other research using third-person subjects also found that the ACE appeared in the front-back direction [37], indicating that other elements in the action sentence beyond the verb do not always integrate into the action simulation. Future research could use eye-tracking technology to record participants’ eye movements during sentence reading or use tasks that include verb aspectual information to further investigate this.

The results of this study also show that the ACE is present in the understanding of both concrete and abstract action sentences in Mandarin. This suggests that the understanding of both concrete and abstract actions may rely on similar psychological simulation processes. Unlike concrete language, which has direct referents in reality, there is still debate about whether psychological simulation exists in the understanding of abstract language. Some studies have confirmed the existence of psychological simulation in both concrete and abstract language [4,23,30]. However, other studies have shown that psychological simulation exists in concrete action sentences but not in abstract action sentences [12,13,31]. In perceptual symbol theory, the relationship between concrete and abstract concepts is bidirectional. Many abstract concepts gain meaning through metaphorical mapping from the concrete domain to the abstract domain, thus making abstract concepts experiential and embodied, just like concrete concepts [8]. Unlike most previous studies that used double-object sentences, this study used Mandarin action sentences as materials, with the verb becoming the core of sentence comprehension. Readers’ understanding of the sentence comes from the body’s perceptual-motor system, and language comprehension involves the participation of the perceptual-motor system, completed through psychological simulation. The experimental results indicate that this is still the case, even for the understanding of abstract action sentences.

In addition, the ACE in Experiments 1 and 2 appeared only in the button responses corresponding to toward the body, which is consistent with previous research findings [38–41]. Recent research has also found that the results of the ACE can sometimes be unstable [42]. This may be due to the fact that, during the evaluation process of the experimental materials, the overall ratings tended to favor sentences with a directionality approaching the body, while sentences indicating movement away from the body were slightly weaker in terms of directional strength. Future studies could expand the number of materials and select sentences with clearer directional cues for further investigation. Furthermore, participants’ focus on different aspects of a sentence may also play a role. Research has demonstrated that the ACE fluctuates depending on variations in font and the use of underlining to highlight specific elements within a sentence, with the matching effect occurring only when the verb is the focal point of emphasis [43]. To deepen the understanding of these effects, eye-tracking or EEG techniques could be used to further explore the impact of aspect markers in Mandarin sentences on constructing psychological simulation, the processes of psychological simulation, and the brain regions involved in this simulation.

The limitations of this study are primarily reflected in the following aspects. First, the study focuses on Mandarin, a specific language type, and only involves particular syntactic markers. Therefore, caution is needed when generalizing the findings to other languages or different types of Mandarin action and aspect markers. Second, although reaction time was used as the outcome measure in this study, the limitations of this method should also be considered. Future research could combine eye-tracking, EEG, and other technologies to further explore the subtle cognitive processes involved in language comprehension. Finally, while the experimental results of this study are reliable in terms of sample size and statistical analysis, the relatively small sample size may affect the robustness and statistical power of the research. Therefore, future studies should increase the sample size and explore additional language types and aspect marker forms.

## Conclusion

This experiment investigated the influence of aspect markers and concreteness/abstractness on the ACE through three behavioral experiments. The results revealed the following:

(1) The aspect marker in Mandarin action sentences did not affect the ACE during the action sentence comprehension process.
(2) The ACE appeared during the comprehension Mandarin both concrete and abstract action sentences.
(3) The comprehension of action sentences in Mandarin may be centered around the psychological simulation of the verb in its general aspect, and this verb simulation is not affected by aspect markers or the concreteness/abstractness of the sentence.

## Supporting information

S1 Appendix The sentence used in the three experiments. (DOCX)

S2 Appendix The English-translated version of the sentence used in the three experiments. (DOCX)
