# Supplementary material for: The influence of aspect markers and tense on the action-sentence compatibility effect in Mandarin action sentence comprehension
Source: PLoS One. 2026 Jan 23;21(1):e0340298. doi: 10.1371/journal.pone.0340298 (PMC12829798; doi:10.1371/journal.pone.0340298)
Supplement: S1 Appendix — (DOCX) [file pone.0340298.s001.docx]

附录

实验1使用的材料

| 现在进行时趋近身体 | 现在进行时远离身体 | 现在完成时趋近身体 | 现在完成时远离身体 |
| --- | --- | --- | --- |
| 小赵正在订购机票  张勇正在收购粮食  李静正在提取货物  张涛正在打开橱柜  小军正在拉开大门  老李正在调整眼镜  小刘正在捡起贝壳  小磊正在接受礼品  韩梅正在拥抱恋人  倩倩正在接听电话 | 小美正在捐赠图书  小海正在递交辞呈  李雷正在运送物品  王伟正在邮递信件  刘伟正在关闭橱窗  小强正在推开大门  张军正在分发传单  王勇正在传递篮球  小芳正在上交试卷  丹丹正在赠送礼物 | 小王已经获得了奖品  李军已经接到了捧花  刘芳已经领取了工资  朱帅已经购买了图书  小陈已经借阅了图书  王静已经拿起了手机  阿杰已经预订了报纸  王涛已经借用了铅笔  老钱已经收受了礼物  老吴已经招收了新生 | 小勇已经支付了现金  王敏已经退还了定金  小娟已经归还了书籍  刘敏已经转交了物品  王丽已经发出了信件  张强已经寄送了快递  张帅已经抛出了捧花  李艳已经撕下了标签  乐乐已经放下了贝壳  张丽已经脱下了外套 |

实验2使用的材料

| 现在进行时趋近身体 | 现在进行时远离身体 | 现在完成时趋近身体 | 现在完成时远离身体 |
| --- | --- | --- | --- |
| 小赵正在博得同情  李静正在争取荣誉  张涛正在借鉴经验  小军正在倾听建议  老李正在收集消息  小刘正在撤回命令  小磊正在收回成本  王磊正在接受任务  韩梅正在采纳意见  倩倩正在得到鼓励 | 王军正在发送消息  小美正在逃避现实  小海正在传达指示  李雷正在传授知识  王伟正在排除杂念  刘伟正在发出命令  张军正在掩饰错误  王勇正在摆脱困境  小芳正在传递消息  丹丹正在拒绝建议 | 小王已经收获了爱情  李军已经吸取了教训  刘芳已经接受了安排  朱帅已经博得了好感  阿杰已经采纳了建议  小雪已经获得了经验  王涛已经接管了政权  王杰已经吸取了经验  老钱已经倾听了意见  老吴已经得到了回报 | 小勇已经泄露了机密  王敏已经传达了要求  小娟已经拒绝了好意  小萱已经拒绝了请求  刘敏已经回避了问题  张强已经丢掉了幻想  张帅已经传授了经验  李艳已经寄托了希望  乐乐已经除去了弊端  张丽已经交代了要求 |

实验3使用的材料

| 具体句趋近身体 | 具体句原离身体 | 抽象句趋近身体 | 抽象句远离身体 |
| --- | --- | --- | --- |
| 老朱将要下载电影  老郑将要接听电话  小杰将要打开橱柜  小兰将要领取工资  小海将要穿上外套  张磊将要拿起手机  丽丽将要拉开大门  晶晶将要预订报纸  小强将要接受礼品  老王将要获得奖品 | 阿超将要寄送快递  张伟将要归还书籍  小美将要放下手机  小莉将要上交试卷  彤彤将要捐赠图书  阿水将要交付租金  浩浩将要退还定金  刘芳将要支付现金  小林将要运送物品  阿杰将要邮递信件 | 张文将要承担责任  小娟将要采纳建议  李静将要采纳意见  小刘将要索取赔偿  莹莹将要收集消息  小英将要接受任务  小天将要聚集力量  小帅将要得到鼓励  阿毛将要借鉴经验  欢欢将要接受安排 | 王敏将要发送消息  张静将要发出指示  李明将要传授经验  张军将要发出命令  刘伟将要传授知识  小霞将要传递消息  小芳将要逃避现实  王静将要奉献青春  老吴将要传达指示  李娟将要传达要求 |
