# Supplementary material for: The influence of aspect markers and tense on the action-sentence compatibility effect in Mandarin action sentence comprehension
Source: PLoS One. 2026 Jan 23;21(1):e0340298. doi: 10.1371/journal.pone.0340298 (PMC12829798; doi:10.1371/journal.pone.0340298)
Supplement: S2 Appendix — (DOCX) [file pone.0340298.s002.docx]

Appendix

The materials used in Experiment 1.

| Present Progressive – Toward the body | Present Progressive – Away from the body | Present Perfect – Toward the body | Present Perfect – Away from the body |
| --- | --- | --- | --- |
| Xiao Zhao is booking a plane ticket.  Zhang Yong is purchasing grain.  Li Jing is retrieving goods.  Zhang Tao is opening the cabinet.  Xiao Jun is pulling open the door.  Lao Li is adjusting his glasses.  Xiao Liu is picking up shells.  Xiao Lei is receiving a gift.  Han Mei is hugging her partner.  Qianqian is answering the phone. | Xiao Mei is donating books.  Xiao Hai is submitting a resignation letter.  Li Lei is delivering goods.  Wang Wei is mailing a letter.  Liu Wei is closing the display window.  Xiao Qiang is pushing the door open.  Zhang Jun is distributing flyers.  Wang Yong is passing the basketball.  Xiao Fang is handing in the test paper.  Dandan is giving a gift. | Xiao Wang has received a prize.  Li Jun has received the bouquet.  Liu Fang has collected her salary.  Zhu Shuai has bought a book.  Xiao Chen has borrowed a book.  Wang Jing has picked up the phone.  Jie has subscribed to the newspaper.  Wang Tao has borrowed a pencil.  Lao Qian has accepted a gift.  Lao Wu has recruited new students. | Xiao Yong has paid in cash.  Wang Min has refunded the deposit.  Xiao Juan has returned the book.  Liu Min has transferred the items.  Wang Li has sent the letter.  Zhang Qiang has mailed the package.  Zhang Shuai has thrown the bouquet.  Li Yan has torn off the label.  Lele has put down the shell.  Zhang Li has taken off her coat. |

The materials used in Experiment 2.

| Present Progressive – Toward the body | Present Progressive – Away from the body | Present Perfect – Toward the body | Present Perfect – Away from the body |
| --- | --- | --- | --- |
| Xiao Zhao is gaining sympathy.  Li Jing is striving for honor.  Zhang Tao is drawing from experience.  Xiao Jun is listening to advice.  Lao Li is gathering information.  Xiao Liu is retracting an order.  Xiao Lei is recovering costs.  Wang Lei is accepting a task.  Han Mei is adopting suggestions.  Qian Qian is receiving encouragement. | Wang Jun is sending messages.  Xiao Mei is escaping reality.  Xiao Hai is conveying instructions.  Li Lei is imparting knowledge.  Wang Wei is eliminating distractions.  Liu Wei is issuing orders.  Zhang Jun is concealing mistakes.  Wang Yong is overcoming difficulties.  Xiao Fang is delivering news.  Dan Dan is rejecting advice. | Xiao Wang has gained love.  Li Jun has learned the lesson.  Liu Fang has accepted the arrangements.  Zhu Shuai has won favor.  Jie has adopted the suggestions.  Xiao Xue has gained experience.  Wang Tao has taken control of power.  Wang Jie has learned from experience.  Lao Qian has listened to the advice.  Lao Wu has been rewarded. | Xiao Yong has leaked the secret.  Wang Min has communicated the requirements.  Xiao Juan has rejected the kindness.  Xiao Xuan has turned down the request.  Liu Min has avoided the issue.  Zhang Qiang has lost hope.  Zhang Shuai has passed on the experience.  Li Yan has placed hope.  Le Le has removed the drawbacks.  Zhang Li has outlined the requirements. |

**The materials used in Experiment 3.**

| Concrete Toward the body | Concrete Away from the body | Abstract Toward the body | Abstract Away from the body |
| --- | --- | --- | --- |
| Lao Zhu is about to download a movie.  Lao Zheng is about to answer the phone.  Xiao Jie is about to open the cupboard.  Xiao Lan is about to receive the salary.  Xiao Hai is about to put on a coat.  Zhang Lei is about to pick up the phone.  Li Li is about to open the door.  Jing Jing is about to order a newspaper.  Xiao Qiang is about to accept a gift.  Lao Wang is about to receive a prize. | Chao is about to send the express delivery.  Zhang Wei is about to return the books.  Xiao Mei is about to put down the phone.  Xiao Li is about to submit the test paper.  Tong Tong is about to donate books.  Shui is about to pay the rent.  Hao Hao is about to return the deposit.  Liu Fang is about to pay in cash.  Xiao Lin is about to transport the goods.  Jie is about to send the mail. | Zhang Wen is about to take responsibility.  Xiao Juan is about to adopt the suggestion.  Li Jing is about to accept the advice.  Xiao Liu is about to demand compensation.  Ying Ying is about to gather information.  Xiao Ying is about to take on the task.  Xiao Tian is about to gather strength.  Xiao Shuai is about to receive encouragement.  Mao is about to learn from experience.  Huan Huan is about to accept the arrangements. | Wang Min is about to send a message.  Zhang Jing is about to issue instructions.  Li Ming is about to impart experience.  Zhang Jun is about to issue an order.  Liu Wei is about to impart knowledge.  Xiao Xia is about to deliver the message.  Xiao Fang is about to escape reality.  Wang Jing is about to dedicate youth.  Lao Wu is about to convey instructions.  Li Juan is about to convey the requirements. |
